# Chikungunya triggers an autophagic process which promotes viral replication

**DOI:** 10.1186/1743-422X-8-432

**Published:** 2011-09-08

**Authors:** Pascale Krejbich-Trotot, Bernard Gay, Ghislaine Li-Pat-Yuen, Jean-Jacques Hoarau, Marie-Christine Jaffar-Bandjee, Laurence Briant, Philippe Gasque, Mélanie Denizot

**Affiliations:** 1 IRG, EA 4517, Immunopathology and Infection Research Grouping, CHR North Felix Guyon and University of La Reunion, St Denis, Ile de la Reunion, France; 2 Centre d'études d'agents Pathogènes et Biotechnologies pour la Santé, CPBS - UMR 5236/CNRS - UM1/UM2, Montpellier, France; 3 Microbiology/Virology Laboratory CHR North Felix Guyon, St Denis, Ile de la Reunion, France

**Keywords:** ChikV, alphavirus, autophagy, innate immunity

## Abstract

****Background**:**

Chikungunya Virus (ChikV) surprised by a massive re-emerging outbreak in Indian Ocean in 2006, reaching Europe in 2007 and exhibited exceptional severe physiopathology in infants and elderly patients. In this context, it is important to analyze the innate immune host responses triggered against ChikV. Autophagy has been shown to be an important component of the innate immune response and is involved in host defense elimination of different pathogens. However, the autophagic process was recently observed to be hijacked by virus for their own replication. Here we provide the first evidence that hallmarks of autophagy are specifically found in HEK.293 infected cells and are involved in ChikV replication.

****Methods**:**

To test the capacity of ChikV to mobilize the autophagic machinery, we performed fluorescence microscopy experiments on HEK.GFP.LC3 stable cells, and followed the LC3 distribution during the time course of ChikV infection. To confirm this, we performed electron microscopy on HEK.293 infected cells. To test the effect of ChikV-induced-autophagy on viral replication, we blocked the autophagic process, either by pharmacological (3-MA) or genetic inhibition (siRNA against the transcript of Beclin 1, an autophagic protein), and analyzed the percentage of infected cells and the viral RNA load released in the supernatant. Moreover, the effect of induction of autophagy by Rapamycin on viral replication was tested.

****Results**:**

The increasing number of GFP-LC3 positive cells with a punctate staining together with the enhanced number of GFP-LC3 dots per cell showed that ChikV triggered an autophagic process in HEK.293 infected cells. Those results were confirmed by electron microscopy analysis since numerous membrane-bound vacuoles characteristic of autophagosomes were observed in infected cells. Moreover, we found that inhibition of autophagy, either by biochemical reagent and RNA interference, dramatically decreases ChikV replication.

****Conclusions**:**

Taken together, our results suggest that autophagy may play a promoting role in ChikV replication. Investigating in details the relationship between autophagy and viral replication will greatly improve our knowledge of the pathogenesis of ChikV and provide insight for the design of candidate antiviral therapeutics.

## Background

Chikungunya Virus (ChikV) is an *Alphavirus *of the *Togaviridae *family transmitted to humans through arthropods bites (mosquitoes of the Aedes genus). First described during a Tanzanian outbreak in 1952 [1], ChikV was recently responsible for a massive re-emerging outbreak in a large tropical area (East Africa and Indian Ocean in 2006, India, Thailand and Indonesia in 2007) and a limited epidemic in Italy in 2007. In 2005-2006, the virus has reached Reunion Island, a south French territory, with an estimated 270 000 cases (1/3^rd ^of the population) and severe forms of the disease, like encephalopathy, in a context of arthralgia, rash, headache and a strong lymphopenia were reported. This 12 Kb positive-strand RNA virus contains two open reading frames (ORFs). The 5' ORF, for the viral replication complex, encodes the non-structural proteins, nsp1, 2, 3, and 4. The 3'ORF, for the structural proteins, encodes for the capsid, envelope glycoproteins (E1 and E2), E3 and 6k proteins. Interestingly, infection with positive-strand RNA viruses may result in the rearrangement of intracellular membranes, constituting scaffolds for viral genome replication [2].

Macro-autophagy, referred herein to autophagy, is a fundamental homeostatic process that leads to the degradation and recycling of long-lived proteins and organelles [3,4]. The molecular machinery of autophagy was identified in yeast by genetic screening with the discovery of 30 AuTophaGy-related genes (ATG) [5]. Most of these genes have now been identified in other organisms as orthologs, suggesting that autophagy is a highly conserved mechanism in eukaryotes [6]. The hallmark of autophagy is the formation of double or multiple membrane-bound vesicles called autophagosomes, which sequester a portion of the cytoplasm and fuse, after maturation, with lysosomes to digest their contents. Formation of the autophagosome requires two ubiquitin-like systems: a conjugate of Atg5-Atg12 and a conjugate in which microtubule-associated protein light chain 3, LC3, is cleaved to produce LC3-I and LC3-II. Autophagy is first a fundamental cell surviving process during starvation conditions but also participates in various processes such as development and tumor suppression [7]. Futhermore, autophagy plays a role in both innate and adaptive immunity in response to pathogens [8]. Indeed, the antiviral action of autophagy has been characterized in infection by sindbis virus, tobacco mosaic virus, vesicular stomatitis virus, herpes simplex virus type 1 and the DNA virus parvovirus B19 [9-11]. In contrast, some viruses have evolved strategies to interfere, escape or even exploit the autophagic machinery. This is the case for certain positive-stranded RNA viruses, such as coronaviruses, picornaviruses, murine hepatitis virus, equine arterivirus, coxsackievirus, hepatitis C virus and dengue virus, which use the autophagosomal machinery to facilitate the assembly of RNA replication complexes [12-15]. More recently, Rodriguez-Rocha et al. found that autophagy-induced in adenovirus infection promotes viral infection and oncolysis [16]. Here, we explore the role of the autophagy machinery in ChikV-infected cells by monitoring the presence of double-membrane vesicles and the distribution of LC3. As well, we demonstrate the effects of perturbing the autophagy pathway, using pharmacological inhibitors or RNA interference, on viral replication. The present study provides the first evidence that cellular autophagy is promoted in ChikV-infected cells and contributes to enhance ChikV replication.

## Materials and methods

### Cells and culture conditions

The HEK.293 cell lines were maintained at 37°C in an humidified atmosphere containing 5% CO2 in complete medium (Dulbecco's modified Eagle's medium) supplemented with 10% heat inactivated fetal calf serum together with penicillin (100 μg/ml), streptomycin (100 U/ml), sodium pyruvate (1 mM), L-glutamine (5 mM) and fungizone (0.5 μg/ml) available from Dutscher (Brumath, France). HEK.293 cells constitutively expressing a GFP-LC3 transgene were generated by transfection of the GFP-LC3 plasmid (kindly provided by M. Biard-Piechaczyk, Montpellier, France). Transfected cells were initially selected with 400 μg/ml of G418 and kept for maintenance with 40 μg/ml of G418.

### Viral stock and inhibition/activation assays

We used a viral isolate (ChikV clone #4) amplified from a patient's serum sample isolated during the 2006 epidemic [17]. One step qRT-PCR was used to amplify the E1 gene.

In infection experiments, biochemical reagents (inhibitor or activator) were added two hours before infection with ChikV (MOI = 1) and samples were analyzed at 24 h and 48 h post-infection.

### Primers used for RT-PCR

The following primers were used:

Hu GAPDH_F, 5'-GAACGGGAAGCTTGTCATCA-3' (position 291-310), and Hu GAPDH_R, 5'-TGACCTTGCCCACAGCCTTG-3' (position 744 -763); sequence reference NM_002046; amplicon 473 bp.

CHIK E1_F, 5'-AAGCTYCGCGTCCTTTACCAAG-3' (position 10387-10400), and CHIK E1_R, 5' -CCAAATTGTCCYGGTCTTCCT-3' (position 10595-10575); sequence reference EU-037962.1; amplicon 209 bp.

### Reagents and antibodies

Rapamycin, an autophagy inducer, was purchased from Sigma, and used at a 1 μM final concentration. 3-Methyl-Adenine, an autophagy inhibitor, was purchased from Sigma, and used at a 10 mM final concentration. Beclin-1 small interfering RNA (siRNA) was purchased from Eurogentec. FDO anti-serum was obtained from a patient with acute Chikungunya, described in (Krejbich-Trotot, et al. 2011 [18]). Monoclonal mouse anti-ChikV antibodies (clones 6C and 4F), a kind gift from Biomerieux, Marcy l'Etoile, France, and IMTSSA (Armed Forces Institute of Tropical Medicine, Pharo), Marseille, France, were used to detect ChikV. The GFP-LC3 plasmid was kindly provided by Martine Biard-Piechaczyk. Anti-Beclin and anti-Actin antibodies were purchased from Sigma.

### Cell immunofluorescence staining

Adherent cells were grown and infected on glass coverslips, fixed and permeabilized at different times post infection by immersion in frozen ethanol for 5 min and conserved at - 20°C. Coverslips were incubated in primary antibodies (1/200) in PBS-BSA 1% and then with secondary antibody conjugated to Alexa594 (1/1000). Nucleus morphology was revealed by DAPI staining (final concentration: 100 ng/ml). Coverslips were mounted in Vectashield H-1000 (Vectorlabs, Clinisciences) and fluorescence was observed using a Nikon Eclipse E2000-U microscope. Images were obtained using the Nikon Digital sight PS-U1 camera system and the imaging software NIS-Element AR.

### Electron micrographs of ChikV-infected HEK.293 cells

Cells infected with Chikungunya virus at MOI = 5 were processed for electron microscopy as described in [19].

### Western blotting

Cells were harvested with a scraper and resuspended in lysis buffer (PBS1X, TritonX100 1%, EDTA 1 mM containing a cocktail of protease inhibitors: PMSF, pepstatin A, leupeptin, aprotinin all at 1 μg/ml final concentration). Protein extracts were mixed with one volume of loading buffer (Tris 0,1 M, Glycerol 10%, SDS 2%) according to Laemmli's protocol. About 50 μg of each sample was loaded onto a 4-12% precasted NuPAGE gels (Invitrogen). After electrophoretic migration, proteins were electrotransferred onto a nitrocellulose membrane (Millipore). Membranes were incubated with primary antibodies and followed by horseradish peroxydase-conjugated secondary antibody. After further washes, the immune complexes were revealed by ECL (PerkinElmer).

### RNA Interference

Cells were grown to 50% to 80% confluence and transiently transfected with an annealed beclin-1 siRNA using FuGEN HD (Roche) according to the manufacter's intructions. An aspecific (unrelated) siRNA was used as a negative control. The silencing efficiency was assessed by Western Blot analyses of whole cell extract using an anti-Beclin-1 antibody. The sense oligonucleotide specific for Beclin-1 was: 5'-CAGUUUGGCACAAUCAAUA-3'.

### Statistics

All values are expressed as means +/- sd and as percentages of 3 independent experiments, each using triplicate culture plates. Comparisons between different treatment regimes have been analyzed by the Mann-Whitney exact test (Graph-Pad, San Diego, CA, USA). Degrees of significance are indicated in the figure captions as follow: * p < 0.05; ** p < 0.01; *** p < 0.001.

## Results

### HEK.293 cells are susceptible to infection by ChikV

**Figure 1:**
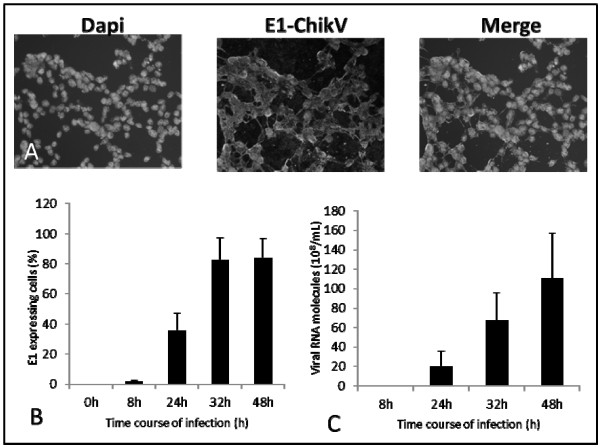
**HEK.293 cells are susceptible to infection by ChikV**. HEK.293 cells cultured on glass coverslips were incubated from 8 h to 48 h with a MOI = 1 of the ChikV or mock infected. **(A) **ChikV infection was monitored by immunofluorescence microscopy 48 h post infection by detection of viral antigens using anti-E1 monoclonal antibody and Alexa Fluor 594-conjugated secondary reagents. Nuclei were stained with DAPI. Magnification = 200× **(B) **The percentage of ChikV-infected cells was quantified during the time course of infection (at least 100 DAPI-stained cells were counted in four separate fields). **(C) **RNA copy number in culture supernatant was determined by qRT-PCR amplification of the E1 gene. A serial dilution of an E1 plasmid was used as a standard. Values are expressed as the mean of five independent experiments +/- standard deviations.

HEK.293 cells were challenged with a multiplicity of infection (MOI) of 1 of the ChikV clone #4 to evaluate their susceptibility to the virus and their ability to produce viral progeny. Susceptibility to ChikV was next determined by mean of immunofluorescence detection of E1 envelope glycoprotein (Figure 1A). Figure 1B shows the percentage of ChikV-infected cells during the time course of infection. At MOI = 1, ChikV antigen E1 was detected on few percentage of cells (1.6% +/- 0.65) as early as 8 hours after infection. About 35% (+/-11%) of the cells were replicating the virus within the first 24 h and about 84% (+/-12%) of the cells were ChikV positive 48 hours post infection. Release of ChikV particles in culture supernatant was monitored by quantification of viral RNA using qRT-PCR (Figure 1C). At 48 h post infection, almost 1.2 × 10^10^/ml viral RNA copies were detected from the cell culture supernatant. These data confirm that HEK.293 cells are susceptible to ChikV and efficiently replicate the virus, as previously reported [20,21].

### Chikungunya virus induces autophagy in HEK.293 cells

**Figure 2:**
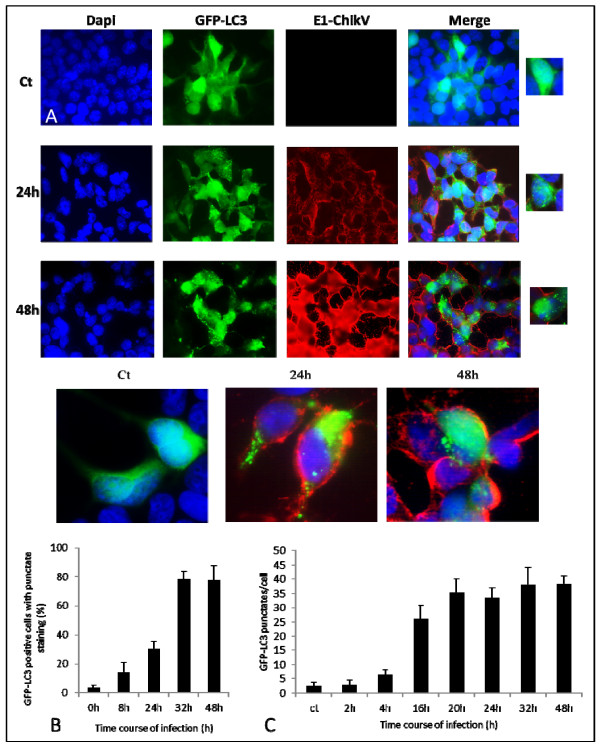
**Redistribution of GFP-LC3 autophagy marker in ChikV-infected HEK.293 cells**. **(A) **GFP-LC3-HEK.293 stable cells cultured on glass coverslips were incubated from 2 h to 48 h with ChikV (MOI = 1) or mock infected (Ct) and analysed by fluorescence microscopy to determine GFP-LC3 distribution. The status of cells regarding infection was determined by detection of ChikV antigens using a monoclonal antibody directed against the E1 glycoprotein and Alexa Fluor 594 (red)-conjugated secondary reagent. Nuclei were stained with DAPI. Magnification = 200× (upper panel), magnification = 600× (lower panel) **(B) **GFP-LC3 positive cells were defined as cells that display more than 5 dots in the cytoplasm. Numbers of GFP-LC3 positive cells were counted on more than 100 cells. **(C) **For each positive cell, the number of GFP-LC3 dots was counted. These results are expressed as mean values obtained from three independent experiments +/- standard errors.

Two forms of LC3 have been reported in the literature. In absence of autophagy, LC3 adopts a diffuse cytoplasmic localization pattern. When autophagy is stimulated, LC3 is conjugated to phosphatidylethanolamine, which participates in the formation of autophagosomes and remains on the autophagosomes membranes [22]. Therefore, modification of LC3 distribution from a diffuse to a punctate staining has been suggested to be a hallmark of autophagy. To decipher the relationship between ChikV and autophagy, we performed fluorescence microscopy experiments in order to follow the LC3 distribution during the time course of ChikV infection. To this end, we generated from HEK.293 cells a stable cell line that expresses the fusion protein of green fluorescent protein (GFP) and LC3. In uninfected cells (Ct), GFP-LC3 showed a diffuse localization throughout the cytoplasm (Figure 2A). During the time course of ChikV infection, GFP-LC3 adopts a punctate staining (number of GFP-LC3 dots per cell in Control: 2.3 +/-1.2; 16 h post infection: 25.9+/-4.9; 48 h post infection: 38.2 +/- 2.8). We showed that both the number of GFP-LC3-HEK.293 cells with punctate GFP-LC3 staining (Figure 2B) and the number of GFP-LC3 dots per cell (Figure 2C) were highly increased. These results indicate that ChikV infection generates a redistribution of LC3 and stimulates autophagy.

### ChikV infection stimulates autophagosome formation in HEK.293 cells

**Figure 3:**
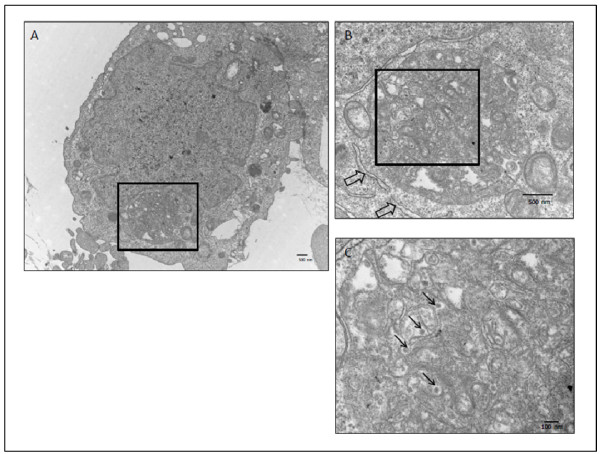
**Electron micrographs of CHIKV-infected HEK.293 cells**. **(A) **Cells infected with ChikV at MOI = 5 were processed for electron microscopy. Infected cells presented an accumulation of membranous vesicles with clear content reminiscent of autophagosomes. A very large vesicle located near the nucleus (see enlargement in **B**) contained degradated material. Smooth reticulum surrounding the vacuole is indicated by arrows. The presence of *bona fide *viral particles with diameters of 40 nm and containing electron dense material detected inside this autophagosome are indicated in **C**.

To confirm that ChikV infection triggers an autophagic process in target cells, we performed an ultrastructure analysis of ChikV-infected HEK.293 cells using transmission electron microscopy (TEM). The cells were challenged with a high infectious dose of ChikV (MOI = 5) to increase the number of infected cells in the culture and favor the visualization of ChikV-positive cells and processed for electron microscopy. In these cells, we observed numerous membrane-bound vacuoles characteristic of autophagosomes (Figure 3A and 3B) that were not present when the HEK.293 cells were mock infected (data not shown). Therefore, this observation confirms that ChikV infection stimulates autophagosome formation in HEK.293 cells. Interestingly, a proportion of virions were observed within the lumen of double-membrane-vesicles (Figure 3C) and we observed a significant proportion of cells that contained assembled *bona fide *viral particles. The colocalisation of viral particles in these structures suggests that they may interfere with the viral life cycle.

### Impairment of autophagy reduces ChikV replication

**Figure 4:**
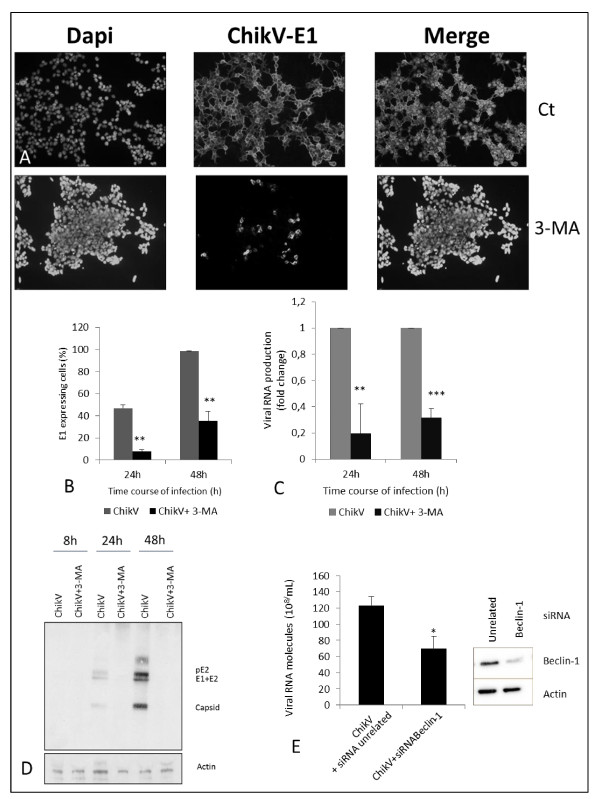
**Effects of autophagic blockade on ChikV infection**. **(A) **HEK.293 cells were pretreated with the inhibitor of autophagy 3-MA (10 mM) for 2 hours or left untreated (Ct). Then, the cells were infected by ChikV at a MOI = 1, as described in Materials and Methods. ChikV infected cells were visualized by immufluorescence, using a monoclonal antibody directed against E1 glycoprotein. Magnification = 200× **(B) **Quantitative analysis of ChikV expression in cells treated or not with 3-MA. **(C) **Consequences of 3-MA treatment on viral particle release. Viral RNA level was determined from cell supernatant. Values are expressed as fold change relative to levels detected from untreated cells. **(D) **Intracellular expression of ChikV structural proteins in HEK.293 maintained in medium alone or supplemented by 10 mM of 3-MA was detected by Western blot. **(E) **HEK.293 cells were infected by ChikV with a MOI = 1 after transfection with Beclin-1 or unrelated siRNA. Expression of Beclin-1 was monitored by immunoblotting using a specific mAb directed against Beclin-1. Quantitative analysis of the viral RNA in cell culture supernatant was determined 24 h post infection. Results are represented as mean values from three independent experiments with standard errors. * P < 0.05; ** P < 0.01; *** P < 0.001.

To determine whether the autophagic process induced during ChikV infection was a host antiviral response or a proviral replication mechanism, we tested the effect of 3-Methyladenine (3-MA) on ChikV replication. 3-MA is a widely used selective inhibitor of autophagy which blocks the formation of autophagosomes and inhibits intracellular protein degradation without affecting protein synthesis [23]. Pre-treatment with 3-MA 2 hours before the infection with ChikV (MOI = 1) resulted in a significant reduction in the number of ChikV infected cells as shown by immunofluorescence microscopy (Figure 4A and 4B). ChikV viral RNA release in culture supernatant was also significantly reduced (Figure 4C). Furthermore, Western Blot analysis showed a limited expression of viral envelope and capsid proteins in infected cells pre-incubated with 3-MA (Figure 4D). This finding suggests that the autophagic machinery may not have an antiviral role during ChikV infection and instead may favor ChikV replication.

To confirm the observation observed with pharmacological inhibitors, we used a target-specific RNA interference approach which will disrupt the autophagosomal machinery. Cells were transfected with siRNA to knock down beclin-1 gene expression. As shown in Figure 4E, cells transfected with specific siRNA reducing cellular Beclin-1 expression poorly supported ChikV replication unlike the unrelated siRNA-treated cells. These results further demonstrate that autophagy is required for effective ChikV replication and enhances an intracellular step of the virus life cycle that remains to be elucidated.

### Induction of autophagy enhances viral growth

**Figure 5:**
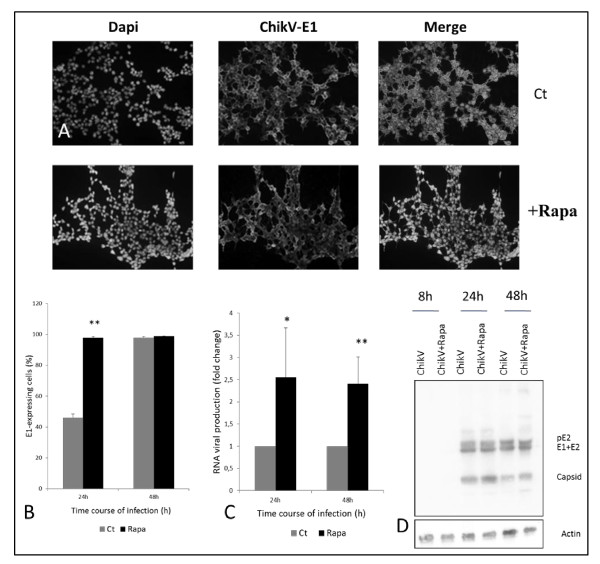
**Effects of autophagic stimulation on ChikV infection**. **(A) **HEK.293 cells were pretreated with the inducer of autophagy Rapamycin (Rapa) (1 μM) for 2 hours before challenge with ChikV (MOI = 1) or left untreated (Ct). Infected cells were detected by immunofluorescence detection of E1 glycoprotein. Nuclei were stained with DAPI. Magnification = 200× **(B) **Quantitative analysis of ChikV infected cells in cultures maintained in the presence of medium alone (Ct) or supplemented with Rapamycin (Rapa). **(C) **Cultures analyzed in (B) were subjected to qRT-PCR quantification of viral RNA present in cell culture supernatant. **(D)**. Expression levels of ChikV structural proteins in HEK.293 infected cells maintained in the presence or absence of Rapamycin was determined by Western blot analysis. Results are expressed as mean values of three independent experiments +/- standard deviations.

To further determine the role of autophagy in viral replication, we investigated the effect of autophagy induction on viral protein expression. Cells were treated with the pharmacological reagent Rapamycin, which has been shown to induce autophagy through inhibition of the mTOR pathway. We observed that pre-treatment with Rapamycin 2 hours before infection with ChikV significantly increased the number of infected cells detected by immunofluorescence detection of E1 glycoprotein (Figure 5A and 5B). Levels of ChikV viral RNA detected in the cell culture supernatant were also increased when cells were treated with Rapamycin (Figure 5C). Furthermore, Western Blot analysis showed that expression of E1 and E2 envelope glycoproteins and capsid protein is enhanced in cells exposed to the drug before viral challenge (Figure 5D). Although we can not totally rule out off-target effects of drugs on processes distinct from autophagy required for viral replication, these results further suggest that the autophagic process enhances viral replication and that it is critical for an intracellular step of the virus life cycle.

## Discussion

In addition to the proteasomal degradation, autophagy represents a major catabolic pathway allowing the turn-over of cytoplasmic constituents in eukaryotic cells. Besides this house-keeping role, autophagy is now considered as a central component of the host immune antimicrobial response against intracellular pathogens. Indeed, certain pathogens, like bacteria, parasites and virus, have been shown to be targeted for autophagic degradation. Therefore some intracellular pathogens have evolved to counteract host autophagy. Three main types of interaction have been described in the literature between autophagy and viruses. (1) Autophagy may have a protective role by limiting virus replication. It has been shown for tobacco mosaic virus, Sindbis virus and parvovirus B19 [9-11]. (2) Autophagic machinery may be disrupted by some viruses. This is the case for herpes simplex virus (HSV-1) which encodes the ICP34.5 protein implicated in autophagy inhibition [24]. (3) Autophagy may have a promoting role for the replication of some viruses. Indeed, the autophagic vesicles are thought to be used as scaffold for intracellular membrane-associated replication factories of RNA viruses [25]. This has been described for coronaviruses, picornaviruses, murine hepatitis virus, equine arterivirus, coxsackievirus and dengue virus [12-15].

The present study explores the role of the autophagic machinery in ChikV-infected cells by monitoring the presence of double-membrane vesicles and the distribution of LC3. We demonstrate the effects of perturbing the autophagy pathway using pharmacological drugs or RNA interference on viral replication. Using these strategies, we provide the first evidence that cellular autophagy is promoted in ChikV-infected cells and that this process enhances ChikV replication.

To understand whether autophagy plays a role in ChikV infection, we assessed the localization of a GFP-LC3 marker in uninfected or ChikV-infected HEK.293 cells by fluorescence microscopy experiments. ChikV infection triggered an increase both in the number of autophagic cells and in the number of GFP-LC3 dots per cell. Of note, accumulation of autophagosomes can be du to an increase in the autophagosomes formation or a decrease of their degradation, which results to the blockade of the fusion with lysosomes. The early induction of autophagy, within 2 hours post infection, is in favour of the first hypothesis, nevertheless we can not exclude that ChikV could also have a role in the late phases of autophagosomes maturation. In the present study, we did not answer this question, which remains to be elucidated.

To confirm our results, we performed an ultrastructure analysis by Transmission Electronic Microscopy and visualized in the cytoplasm of ChikV-infected cells numerous vacuoles with features of autophagosomes. Furthermore, virions are localized in the lumen of those structures, consistent with the hypothesis that ChikV may recruit autophagosomes into virus factories to generate a scaffold for replication complex. This hypothesis need to be further investigated. The next step of the study was to investigate the impact of the autophagic process triggering in ChikV replication. For this purpose, we blocked the autophagic process by two ways: a pharmacological inhibition, using a common inhibitor, 3-Methyladenine, and a genetic inhibition, using RNA interference against the transcript of the protein Beclin 1. We identified that ChikV replication is dramatically decreased by both approaches, attesting that the autophagic machinery is needed for ChikV replication. Both the intracellular infection and extracellular RNA viral load were assessed and were found to be affected, suggesting that the autophagic machinery is needed to promote virus replication inside the cell and not only for the release of new ChikV progeny virions outside the cell. Of note, the partial blockade of both infection and replication suggests that autophagy contributes but is not strictly necessary for ChikV replication. We next used an autophagic inducer, Rapamycin, to decipher the impact of a stimulation of the autophagic process on ChikV replication. After treatment with Rapamycin, cells were much more permissive or sensitive to ChikV infection, suggesting that autophagy is a promoting factor for ChikV replication.

Our previous study provided evidence that completion of the apoptotic process is an important element for efficient virus propagation [18]. Furthermore, ChikV-induced apoptosis possibly leads to the persistence of the virus in macrophages, and hence shielded from the immune system. On the way to decipher the mechanisms either controlling viral infection or, on the contrary, promoting viral spreading and pathogenicity, it seems that this virus has evolved to control both the apoptotic and the autophagic process. ChikV seems to be more than opportunistic and can exploit the classic cellular immune response. From a therapeutic standpoint, available drugs controlling autophagy could be used to limit ChikV spreading.

## Nonstandard abbreviations

ChikV: Chikungunya Virus; LC3: light chain 3; GFP-LC3: LC3 fused to GFP; 3-MA: 3-methyladenine; Rapa: Rapamycin.

## Competing interests

The authors declare that they have no competing interests.

## Authors' contributions

MD designed research; MD, GLPY, PKT, BG and JJH performed research; MD, LB and PG and MCJB analysed data; MD wrote the paper. All authors read and approved the final manuscript.
